# Poplar: a phylogenomics pipeline

**DOI:** 10.1093/bioadv/vbaf104

**Published:** 2025-05-06

**Authors:** Elizabeth Koning, Arjun Subedi, Raga Krishnakumar

**Affiliations:** Biosecurity and Bioassurance Department, Sandia National Laboratories, Livermore, CA 94550, United States; Department of Computer Science, University of Illinois, Urbana-Champaign, Urbana, IL 61801, United States; Biosecurity and Bioassurance Department, Sandia National Laboratories, Livermore, CA 94550, United States; Department of Computer Science, California State University, Fullerton, CA 92831, United States; Biosecurity and Bioassurance Department, Sandia National Laboratories, Livermore, CA 94550, United States

## Abstract

**Motivation:**

Generating phylogenomic trees from the genomic data is essential in understanding biological systems. Each step of this complex process has received extensive attention and has been significantly streamlined over the years. Given the public availability of data, obtaining genomes for a wide selection of species is straightforward. However, analyzing that data to generate a phylogenomic tree is a multistep process with legitimate scientific and technical challenges, often requiring a significant input from a domain-area scientist.

**Results:**

We present Poplar, a new, streamlined computational pipeline, to address the computational logistical issues that arise when constructing the phylogenomic trees. It provides a framework that runs state-of-the-art software for essential steps in the phylogenomic pipeline, beginning from a genome with or without an annotation, and resulting in a species tree. Running Poplar requires no external databases. In the execution, it enables parallelism for execution for clusters and cloud computing. The trees generated by Poplar match closely with state-of-the-art published trees. The usage and performance of Poplar is far simpler and quicker than manually running a phylogenomic pipeline.

**Availability and implementation:**

Freely available on GitHub at https://github.com/sandialabs/poplar. Implemented using Python and supported on Linux.

## 1 Introduction

Creating phylogenetic and phylogenomic trees from sequenced genomes is core to most areas of bioscience and biotechnology, as an understanding of the genomic relationships between organisms is critical for prevention, surveillance, intervention, and optimization of biological processes (Kapli *et al.* 2020). In particular, understanding microorganismal diversity is critical for defending against pathogens and for optimizing organisms to support the bioeconomy, with examples including bacteria, fungi, and even members of the plant kingdom such as algae (Spatafora *et al.* 2017, Navarro *et al.* 2021, Azouri *et al.* 2021, Nakano *et al.* 2023). In addition, phylogenetics plays a critical role in viral, bacterial, and fungal pathogen and pandemic monitoring, as exemplified recently by SARS CoV2 and fungal plant pathogens (Turakhia *et al.* 2022, Ki and Terhorst 2022, De Maio *et al.* 2023, Dort *et al.* 2023).

To date, there are numerous strategies and methods associated with building these trees. During this process, it is essential to combine biological meaning with computational efficiency and flexibility. While early approaches relied on observable physical and metabolic characteristics, the expansion of genetics and genomics over the decades has added a significant amount of resolution to these methods (Hug *et al.* 2016).

Building a species tree requires genomic data from each included species to be sequenced, analyzed, compared, and then constructed into a tree. The specific steps may differ, but a typical pipeline includes sequencing, identifying homologs, aligning sequences, inferring gene trees, and finally constructing a species tree (Young and Gillung 2020). Each step requires one or more software tools requiring various inputs and data formats. In addition, software that offers sequence-to-tree analysis has its own limitations.

For example, MEGA is a comprehensive tool for tree-building (Tamura *et al.* 2021). However, MEGA requires either a genome annotation or whole genome multiple genome alignment, and while this can be extremely helpful for some applications, it can have limitations in other situations, for example, if users want to focus on specific gene subsets without requiring significant manual intervention, which might be challenging for nonsubject matter experts. Another challenge a user may face while attempting to use whole genomes is the requirement of a single sequence for each species, which excludes assemblies of scaffolds. If there is an annotation, the user must use another tool to group gene sequences before alignment. Furthermore, MEGA is not open source, so users cannot adapt it or run on incompatible operating systems. It also does not support parallelism.

EasyCGTree is another tool for inferring phylogenetic trees (Zhang *et al.* 2023). It includes options for multiple inference methods and does include a step to group sequences to form gene trees. However, it does not include parallelism, which is a significant roadblock when building increasingly larger trees.

A third software tool for tree inference is Read2Tree (Dylus *et al.* 2024). While the authors of Read2Tree report highly accurate output trees, Read2Tree requires an internet connection during its run as well as a download from the author’s Orthologous Matrix (OMA) database before running. For some applications, the required remote connection would be a security concern or a practical restriction. As for the data required from the external database, the database does not include sequences for all species. Although we have not done a comprehensive examination of the included species, in our comparative testing, we found OMA includes very few fungal sequences. Read2Tree also uses reads, rather than assemblies. In some cases, this may be very useful, but if genomes are selected from the National Center for Biotechnology Information (NCBI) database, they will typically have assemblies available, which allows for downloading and managing a much smaller amount of data. In addition, reads can be assembled into genomes or transcriptomes, but the reverse is not true.

To address the issues of ease of use, parallelism and flexibility outlined above, we present Poplar, a novel software tool for the pipeline from assembled genome to phylogenomic tree. As a tree species, “poplar” connects the concept of biological trees with the phylogenomic trees the software infers. Specifically, poplar trees have a reputation for fast growth, and Poplar, the software tool, is designed to quickly grow a phylogenomic tree (The Editors of Encyclopaedia Britannica 2023). Tasks as seemingly simple as converting file types or as time-consuming as genome annotation can bog down the development of a phylogenomic inference pipeline, and Poplar addresses the issue by smoothing the connections between the numerous steps in tree inference and using annotated or unannotated genomes.

## 2 Background

To obtain a species tree from a collection of assembled genomes typically requires a variety of software packages. Although each step in the pipeline has well-established and ever-improving tools, those tools have unique file formats and input requirements that are frequently incompatible with other tools. Many steps have entirely independent computation, but executing in parallel requires manually initiating parallel jobs. Poplar addresses these concerns and manages converting file types, renaming sequences, and running tasks in parallel.

The key steps are:

1. Assemble genome from reads (if using next-generation sequencing data as opposed to a preassembled genome)
2. Identify gene sequences
3. Group sequences into genes across species
4. Align gene sequences
5. Create gene trees
6. Merge gene trees into species tree

There may be variation in these steps, many of which are described in Kapli *et al.* (2020). Some approaches will require whole genome alignment (WGA) rather than perform alignments for gene sequences, for example. WGA is done through specialized tools such as Cactus (Armstrong *et al.* 2020) or more general alignment tools such as RAxML-NG and Muscle (Kozlov *et al.* 2019, Edgar 2022). WGA is computationally intensive and therefore larger alignments are often not feasible on computational resources that are readily available to most researchers. Kmer-based distance methods can also substitute for alignment, and they bypass using full gene sequences, but rather use dictionaries of kmers. These methods are extremely helpful for fast examination of relationships between genomes, particularly large numbers of large genomes (Nasko *et al.* 2018, Manekar and Sathe 2018, Moeckel *et al.* 2024). However, the caveat of using kmer-based methods is that the gain in speed sacrifices some accuracy, and although this loss in accuracy can be mitigated by having more individual genomes to analyze (i.e. more pairwise distance comparisons can make for a more accurate overall tree), we focused Poplar to use methods (discussed below) that are more resilient and accurate even with low genome/sequence numbers.

Within gene-driven trees are a specific subset that are frequently used—alignment based on ribosomal genes. Although there are numerous examples of this in the literature, a significant one is the recent novel view of the tree of life, where multiple ribosomal genes were used (Hug *et al.* 2016). Although focusing on ribosomal genes can standardize comparisons, it can be limiting in cases needing discernment of similar organisms, or strains within a species, where the ribosomal genes are likely to be very similar and may not provide the desired resolution.

Most often, phylogenomic trees are created through *ad hoc* pipelines, such as in the 1000 Plants Initiative and the Avian Phylogenomics Project (Wickett *et al.* 2014, Jarvis *et al.* 2014). This has the benefit of allowing the scientists to use tools they expect to be the most suitable for their data in each step, but it also requires implementing many elements of the application, including the tools to connect the main steps. *Ad hoc* pipelines also face challenges in presentation. The methods to construct published trees are often not specific enough for other researchers to adapt or reproduce. As code for the pipelines are often not included with publications, each research group must implement their own pipeline, which may or may not share characteristics with other pipelines, leading to wasted energy and a lack of clarity in the origins of various phylogenomic trees. In designing Poplar, our goal is to implement a tried-and-tested combination of the algorithms required to complete the key steps outlined below. We acknowledge that there are alternatives, and ultimately the long-term goal of Poplar is to be modular and allow for flexibility in algorithm usage; however, we describe here a pipeline that uses established tools in an efficient and accessible way. Specifically, we focus on optimizing a few processes outlined below.

Inferring gene trees requires collections of gene sequences. Identifying genes as genes that occur across multiple species requires a genome annotation. *De novo* gene prediction identifies probable genes based on patterns within the genomic sequence, while alignment-based methods rely on a reference database to compare the sequences in a genome with the known genes (Vallender 2009). Either approach is a significant task in terms of both labor and computation. On the other hand, identifying homologs that do not have an identified function is a simpler task, and provides far less information. They may resemble one another due to shared ancestry or horizontal gene transfer, or the sequences may have no relation. Occasionally, this task is done with grouping algorithms such as DBSCAN (Density-Based Spatial Clustering of Applications with Noise) (Edla and Jana 2012). If an annotation is available for some genomes of interest but not for others, that typically means that the unannotated genomes will be used for all included species.

As described by Kapli *et al.* (2020), gene tree inference may be done through techniques including distance methods, such as Neighbor Joining, or character-based methods, such as Maximum Likelihood. Deciding which method is most practical for a particular situation is dependent on factors such as how closely related the sequences are.

Given a set of gene trees, various supertree methods also exist to infer the species trees. A popular tool for species tree inference is ASTRAL-Pro3 (Zhang and Mirarab 2022b), which optimizes for the number of quartets occurring in the gene trees in the resulting species tree. Poplar incorporates ASTRAL-Pro3 as a method for fusing the gene trees generated in the previous step. We selected ASTRAL-Pro3 specifically to allow for the use of multicopy gene trees, which removes the need to choose the best version of mutlicopy genes within a genome and thereby facilitating automation and reducing bias. For users who want an alternative, Poplar also allows the use of ASTRAL-IV, which is designed particularly for single-copy input, and ASTRAL-Pro3 is designed for multicopy input (Zhang and Mirarab 2022a).

## 3 Algorithm

**Figure 1.**
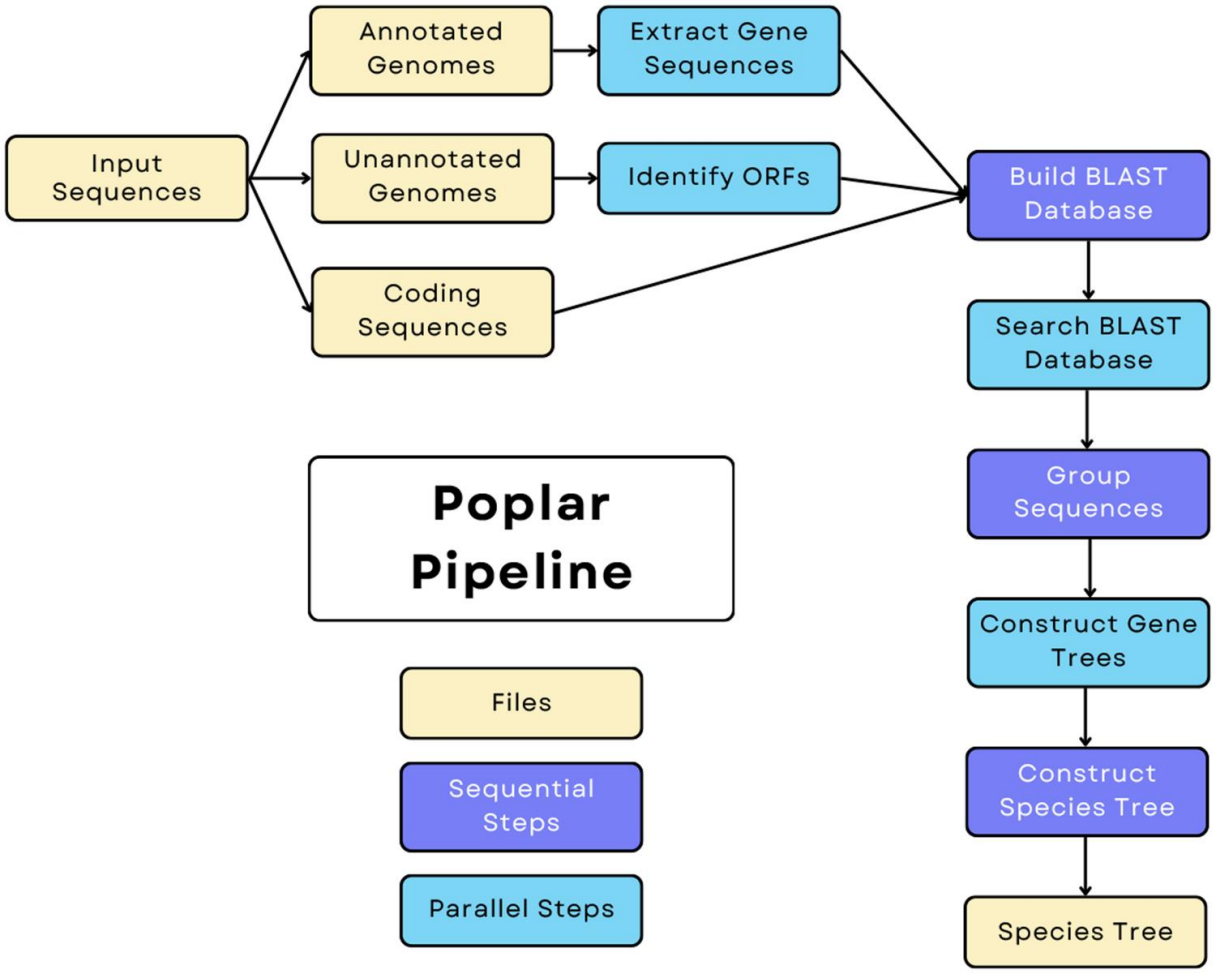
Illustration of the Poplar pipeline from input sequence to species tree.

Poplar provides a structure to connect established tools for each step of phylogenomic tree inference. Beginning from gene or genome data, it works through identifying relevant sequence, building possible gene groupings, inferring gene trees, and constructing a species tree. Figure 1 illustrates the pipeline.

Poplar does not have a next-generation sequencing analysis or genome assembly module, and the user provides either genomes, scaffolds, or genes. We will describe the other key steps identified in the background section which are implemented by Poplar.

### 3.1 Identification of gene sequences

The first step in Poplar is identifying gene sequences for each input species. For some species, these will be input as sequences, and for others, the sequences must be extracted from the genome scaffold sequences. If coding sequences are provided for a species, those sequences will be prioritized. Otherwise, if an annotated genome with identified genes is provided, then the sequences are extracted and used as potential genes. If not, then Open Reading Frames (ORFs) are extracted from the genome using orfipy (Singh and Wurtele 2021). In a single run of Poplar, each type of data may be used for different genomes. Currently, the implementation requires nucleotide sequences.

### 3.2 Sequence grouping into genes across species, and alignment of gene sequences

Following the identification of genes and ORFs, Poplar groups the sequences in clusters.

First, Poplar uses Nucleotide BLAST database to identify similar sequences across genomes. All the sequences are collected in a Nucleotide BLAST database (Altschul *et al.* 1990). Poplar then queries the database for each sequence. The default similarity threshold is an expectation value (E value) of $1^{-20}$; however, this threshold may be increased or decreased by the user if the input organisms are less or more closely related, respectively.

The results of the searches are used to create a distance matrix, which is then fed into an implementation of Density-Based Spatial Clustering of Applications with Noise (DBSCAN) for grouping (Ester *et al.* 1996, Schubert *et al.* 2017). DBSCAN was selected for this step because it does not force a fixed number of groups, a fixed size of groups, or all sequences to be placed into a group. Instead, it identifies dense clusters as sequences that it declares groups. After using DBSCAN to create these groups, Poplar omits groups that are too small or too large, based on user input. By default, the minimum group size is three, and the maximum group size is 100.

Using a grouping algorithm like DBSCAN to identify homologs across identified gene sequences is not the most popular way that gene clusters are identified. Often, orthologs will be identified and stored in a database such as OMA for future reference (Vallender 2009). Using the BLAST search followed by DBSCAN only considers the similarity between two sequences, and not any contextual information about their respective genomes. Yet, DBSCAN has been used for identifying gene clusters, including as an aspect of the tool GeneGrouper (McFarland *et al.* 2022). The results of Poplar also demonstrate the effectiveness of using a distance-based clustering for sequences.

Rather than rely on an external database or an intensive annotation process, Poplar considers a large set of sequences, and identifies those that are most similar. The BLAST database is stored locally, allowing Poplar to execute offline. The pairing of BLAST and DBSCAN allows Poplar to identify similarities between sequences, including sequences from genomes that have not been annotated.

The output of the sequence grouping step is the sets of sequences identified as similar.

### 3.3 Creation of gene trees

Once Poplar has a set of sequence groups, it selects a subset of the groups to build gene trees. By default, it will randomly select 50 groups, and a user may specify some other maximum number of trees to build.

Poplar infers gene trees from the sequence groups via MAFFT (Katoh and Standley 2013) for sequence alignment and RAxML-NG (Kozlov *et al.* 2019) for gene tree construction. By default, Poplar also uses the default settings for both tools. The user may select the maximum number of gene trees to create, as the number of groups available to create trees is likely to be high, due to the nature of the sequence grouping process. MAFFT and RAxML-NG are both established tools in the field. RAxML-NG outputs the best tree for each set of sequences, which is then the input for creating the species tree.

### 3.4 Merging of gene trees into species trees

To infer a species tree from a set of gene trees, Poplar uses ASTRAL-Pro3 (Zhang and Mirarab 2022b). Like MAFFT and RAxML-NG, ASTRAL is an established and popular tool. The output of ASTRAL is a single tree including each of the input species. The branch lengths are derived using ASTRAL-Pro3’s branch length estimation in the gene trees (Tabatabaee *et al.* 2023), and then propagated through the species trees using RAXML.

## 4 Implementation

Poplar works as a framework or backbone to connect various well-established tools. It runs as a Python script that manages inputs and refers to other tools, allowing the user to select options such as the number of gene trees, the maximum number of sequences to include in a gene group, and the E value threshold for BLAST. To manage parallelism, it uses Parsl (Babuji *et al.* 2019), a Python workflow library. Parsl allows Poplar to fluctuate the hardware allocated to the tasks as needed.

Our tests were performed on a system using Slurm; however, Parsl also supports running a workflow locally, or via other schedulers or cloud services. Changing the type of system used is done in a configuration file, and a Parsl “provider” is selected. The configuration file must be customized for the system being used in order for the Parsl to use the correct Slurm queue or other method of accessing the hardware.

Some steps required new code within Poplar. Most of the new code involves changing file formats and data labels, and are clearly outlined in our user manual. The one more substantial element is in identifying homolog groups using DBSCAN, which are then used for the “gene” trees. The algorithm cannot confirm that the groups represent a single gene across the species. Rather, it merely groups similar sequences together. However, as we will demonstrate in the Results, and based on prior literature, this is an effective method for finding groups of sequences that can be annotated as individual genes for tree-building (McFarland *et al.* 2022).

Not using new, untested algorithms is a strength of the current iteration of Poplar—because it does not utilize any new ways of generating gene trees or species trees, the end result when using Poplar is the direct result of using MAFFT, RAxML, and ASTRAL-Pro, which are all trusted software. Poplar streamlines the interface between these reliable tools. When a new software is introduced or different tools and models are needed for particular uses, Poplar can also be updated to use the most accurate algorithms. The accuracy is that of its component tools, allowing the user to know what to expect of the results in terms of theoretical guarantees and experimental performance. Poplar uses a configuration file to allow users to specify the options for each tool.

The input to Poplar is FASTA files. As discussed earlier, these may be coding sequences, annotated genomes, or unannotated genomes. The intermediate data from each of these steps may be preserved for user reference or be deleted to minimize stored files. Poplar also supports check-pointing through Parsl. The clustering algorithm outputs lists of sequence identifiers for all groups it creates. MAFFT outputs an alignment file in FASTA format, and RAxML outputs gene trees in Newick format. The output of Poplar is a Newick tree, including branch lengths.

## 5 Results

### 5.1 Testing data

The testing data came from NCBI, Joint Genome Institute (JGI)’s MycoCosm, and JGI’s PhycoCosm (Table 1). Specifically, fungal genomes were chosen due to the fact that the fungal kingdom is both highly diverse and rapidly growing, and the use of phylogenetic trees to characterize new and emerging fungal species is key and widespread (Frąc *et al.* 2018, Naranjo-Ortiz and Gabaldón 2019, Shumskaya *et al.* 2023). The test datasets are:

**Table 1.** Description of input datasets.

Dataset	Number of genomes	Data types	Average genome size (bp)	Average number of genes
*Kickxellomycotina*	13	Unannotated, annotated, coding sequences	37 359 022	8551
*Mortierellomycotina*	88	Coding sequences	41 292 989	11 837
*Rhodophyta*	26	Coding sequences	31 174 001	7111
NCBI commonly studied organisms	20	Coding sequences	955 Mb	24 012
*Spermatophyta*	13	Coding sequences	1 010 911 614	41 415

1. The 13 *Kickxellomycotina* genomes available through MycoCosm (Chang *et al.* 2015, Wang *et al.* 2016, Mondo *et al.* 2017, Ahrendt *et al.* 2018, Amses *et al.* 2022, Reynolds *et al.* 2023).
2. The 88 *Mortierellomycotina* genomes available through MycoCosm (Mondo *et al.* 2017, Uehling *et al.* 2017, Vandepol *et al.* 2020, Mesny *et al.* 2021, Chang *et al.* 2022, Mathieu *et al.* 2024).
3. The 26 *Rhodophyta* coding region sets available through PhycoCosm (Nozaki *et al.* 2007, Bhattacharya *et al.* 2013, Collén *et al.* 2013, Nakamura *et al.* 2013, Schönknecht *et al.* 2013, Brawley *et al.* 2017, Lee *et al.* 2018, Rossoni *et al.* 2019, Nakamura-Gouvea *et al.* 2022, Cho *et al.* 2023).
4. NCBI’s 20 commonly used organisms for molecular research projects (Assembly 2009, 2013a,b, 2014a,b, 2015, 2016a, 2017, 2018a,b, 2019a,b,c, 2020a,b, 2021, 2022a, 2023a,b, 2024a).
5. The five genomes of *Puccinia graminis* available through MycoCosm (Duplessis *et al.* 2011, Li *et al.* 2019).
6. Thirteen plant species from *Spermatophyta* with coding sequences available through NCBI. Species: *Arabidopsis thaliana*, *Populus alba x Populus*, *Lithocarpus litseifolius*, *Quercus lobata*, *Ensete ventricosum*, *Pisum sativum*, *Miscanthus lutarioriparius*, *Persea americana*, *Acer negundo*, *Lactuca saligna*, *Prunus dulcis*, *Theobroma cacao*, and *Cajanus cajan* (Assembly 2016b,c,d, 2019d,e, 2020c,d, 2022b,c, 2023c,d,e, 2024b).

For the datasets from JGI, the reference trees came from the JGI webpage. For the NCBI dataset, the reference tree is from the species classifications. The reference trees do not include branch lengths. Branch lengths were not included on the reference trees or in the accuracy metrics, even in the trees where they are available.

A key criterion in selecting the datasets was the availability of a fully resolved reference tree from clear and well-grounded methodology. JGI publicly describes their Annotation Pipeline, and all included genomes are presented in the species tree, including where there are multiple genomes from a single species. NCBI presents only the classification of species, which means the tree has many polytomies. Polytomies are not an issue with the 20 common species as they are distantly related but are an issue with any closely related set of species or any species with multiple variants.

### 5.2 Metrics

We report the accuracy of trees by the Robinson-Foulds (RF) distance (Robinson and Foulds 1981) as well as triplet distance (Sand *et al.* 2013, 2014). The RF metric works by identifying all bifurcations of the trees, and comparing the bifurcations in one tree with those of the other. If a split is present in only one of the two trees, the distance score increases by 1. The reported distances are normalized to be out of 100%, where 0% is an identical tree and 100% shares no bifurcations. The triplet distance measures the differences in topologies of all subsets of three leaves within the trees to compare two rooted trees (a similar metric, the quartet distance, exists for unrooted trees).

Where the reference tree is not a binary tree, we do not report the additional splits in the generated trees as inaccuracies. MycoCosm presents binary trees, while NCBI provides the classifications of the species, which are not binary trees but also do not assert that there are not additional relationships that are not captured by the classification schema. Poplar reports binary trees.

All tree figures were visualized using online tree viewer developed by Huerta-Cepas et al. (2016).

### 5.3 Kickxellomycotina

**Figure 2.**
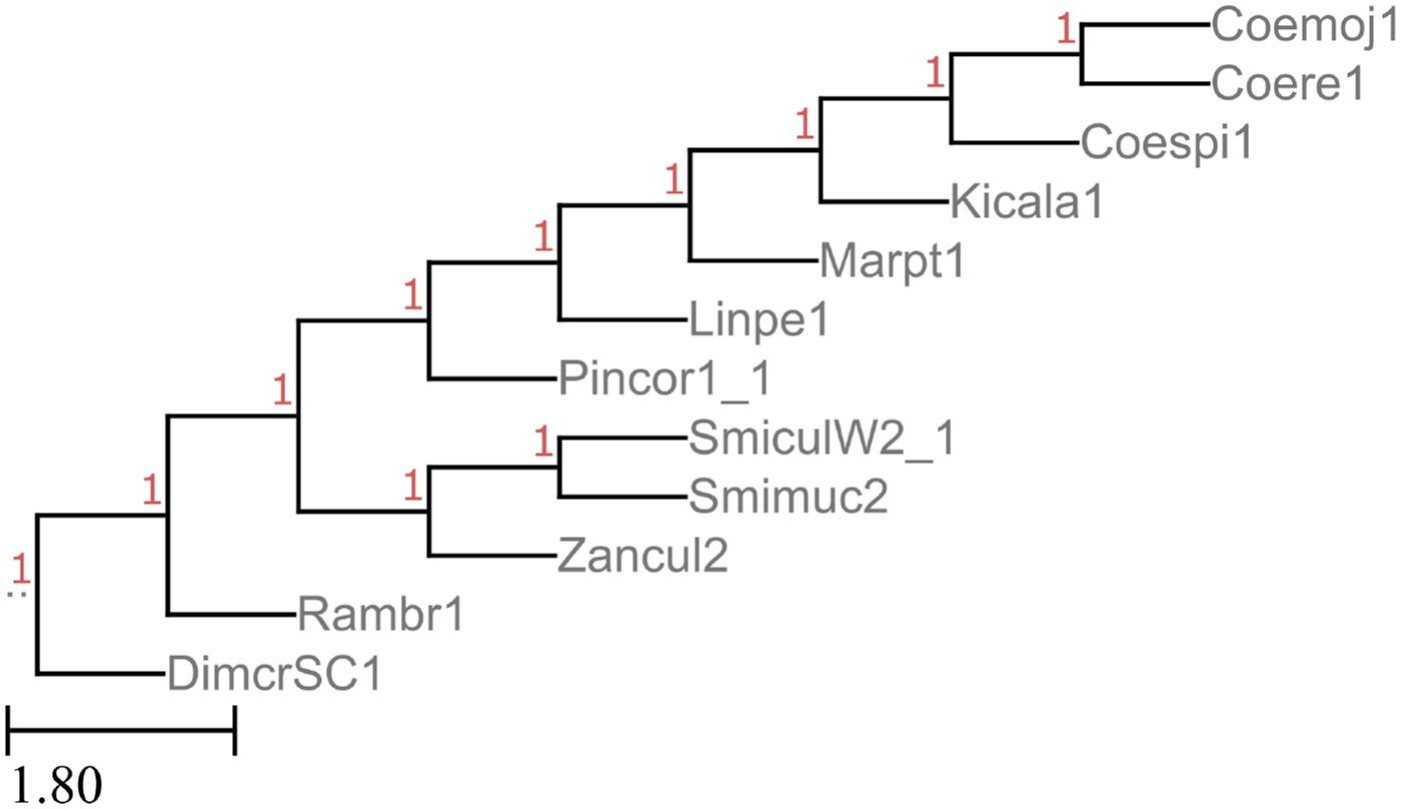
Reference tree of *Kickxellomycotina* genomes, according to JGI’s MycoCosm.

**Figure 3.**
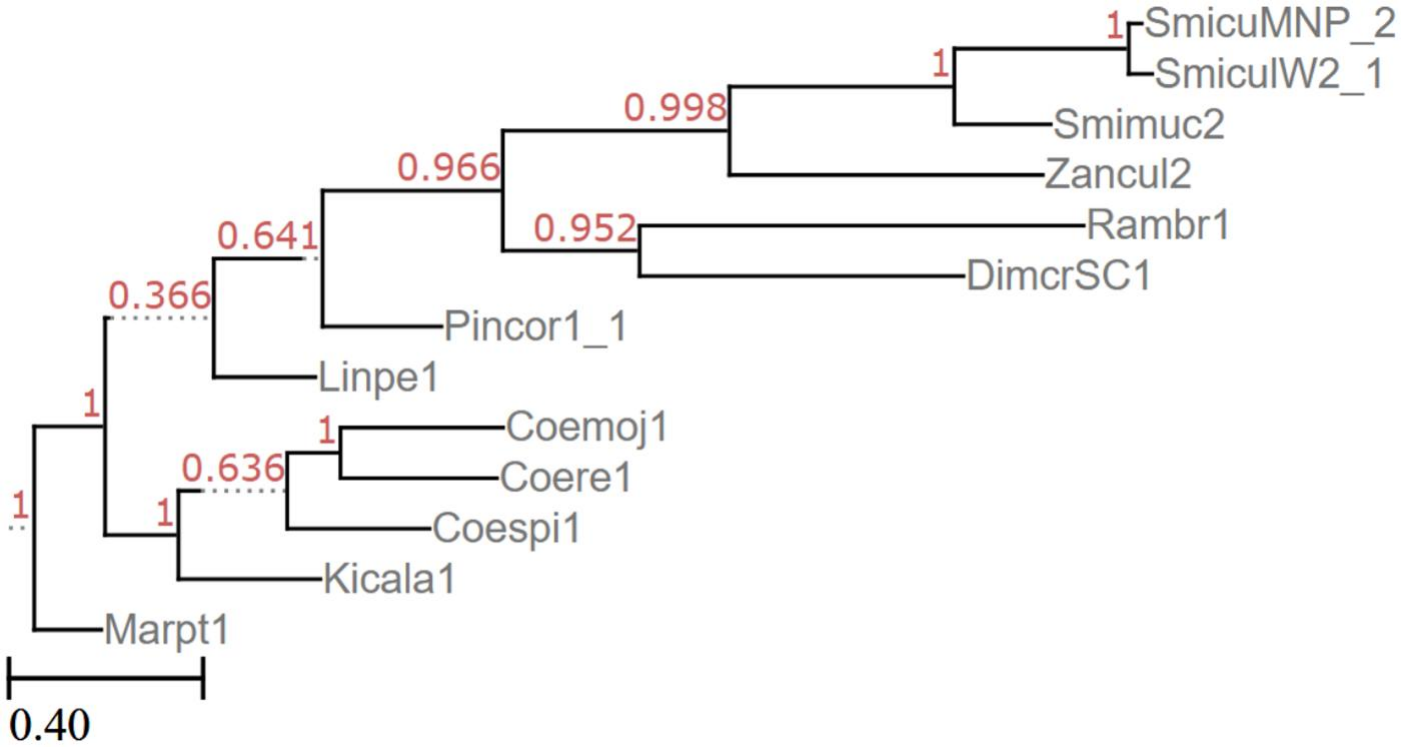
Poplar generated tree of *Kickxellomycotina* genomes with triplet error rate of 0.594, according to one run of Poplar with 50 gene trees given the coding sequences.

The *Kickxellomycotina* classification within fungi has 13 genomes in JGI’s MycoCosm, with the tree shown in Fig. 2. Some species within *Kickxellomycotina* have multiple genomes represented. For our test, we compared the use of unannotated genomes, coding sequences, and fully annotated genomes (as per the GFF3 found on the JGI website). Each of the tests were run 5× with either 50, 100, or 500 gene trees, and performance was compared across these runs, as shown in Tables 2–4 and 7–15. We notice that both for triplet error and RF error, the rates are similar for the unannotated and annotated genomes, suggesting that annotation is not required for accuracy (Tables 2–4). Also, we note that the error rate in between trees generated by Poplar is comparable with the error rate between Poplar trees and the gold-standard tree downloaded from JGI. This suggests that the error rates observed fall within the stochasticity of tree-building by the underlying algorithms, and that Poplar is not introducing any additional error. Finally, we see that adding gene trees tends to improve the error rates, consistent with what is reported in the literature (Szöllősi *et al.* 2015, Yan *et al.* 2023). We therefore believe that misplacement of individual taxa for the most part can be mitigated by adding more gene trees. The results of one run with a 11% RF error rate is shown in Fig. 3.

**Table 2.** Triplet distance and Robinson-Foulds distance of Poplar-generated *Kickxellomycotina* trees from coding sequences.^a^

Input type	Number of gene trees	Number of runs	Average triplet error rate (intra-Poplar)	SD triplet error rate (intra-Poplar)	Average triplet error rate (Poplar-to-reference)	SD triplet error rate (Poplar-to-reference)	Average RF error rate (intra-Poplar)	SD RF error rate (intra-Poplar)	Average RF error rate (Poplar-to-reference)	SD RF error rate (Poplar-to-reference)
*Kickxellomycotina* CDS	50	5	0.479	0.3544	0.6700	0.1439	0.11	0.0852	0.10	0.0707
*Kickxellomycotina* CDS	100	5	0.3494	0.1267	0.5908	0.1396	0.11	0.0852	0.10	0.0707
*Kickxellomycotina* CDS	500	5	0.5462	0.3257	0.5308	0.3057	0.10	0.0649	0.08	0.0837

a Distances shown are from the reference and from each other upon five iterations.

**Table 3.** Triplet distance and Robinson-Foulds distance of Poplar-generated *Kickxellomycotina* trees from unannotated genomes.^a^

Input type	Number of gene trees	Number of runs	Average triplet error rate (intra-Poplar)	SD triplet error rate (intra-Poplar)	Average triplet error rate (poplar-to-reference)	SD triplet error rate (poplar-to-reference)	Average RF error rate (intra-Poplar)	SD RF error rate (intra-Poplar)	Average RF error rate (Poplar-to-reference)	SD RF error rate (Poplar-to-reference)
*Kickxellomycotina* Unannotated genome	50	5	0.3001	0.1666	0.6328	0.1176	0.17	0.0470	0.12	0.0837
*Kickxellomycotina* Unannotated genome	100	5	0.5754	0.3892	0.6292	0.1347	0.18	0.1508	0.20	0.1225
*Kickxellomycotina* Unannotated genome	500	5	0.2631	0.2697	0.3624	0.3156	0.00	0.00	0.10	0.00

a Distances shown are from the reference and from each other upon five iterations.

**Table 4.** Triplet distance and Robinson-Foulds distance of Poplar-generated *Kickxellomycotina* trees of annotated genomes.^a^

Input type	Number of gene trees	Number of runs	Average triplet error rate (intra-Poplar)	SD triplet error rate (intra-Poplar)	Average triplet error rate (Poplar-to-reference)	SD triplet error rate (Poplar-to-reference)	Average RF error rate (intra-Poplar)	SD RF error rate (intra-Poplar)	Average RF error rate (Poplar-to-reference)	SD RF error rate (Poplar-to-reference)
*Kickxellomycotina* GFF3	50	5	0.5198	0.2403	0.5818	0.1662	0.28	0.2707	0.28	0.1789
*Kickxellomycotina* GFF3	100	5	0.5265	0.1987	0.4552	0.2355	0.10	0.0649	0.06	0.0548
*Kickxellomycotina* GFF3	500	5	0.4663	0.2280	0.5578	0.2935	0.08	0.1005	0.12	0.0447

a Distances shown are from the reference and from each other upon five iterations.

**Table 5.** Triplet and RF error rates for *Mortierellomycotina* and *Rhodophyta*.^a^

Input type	Average triplet error rate (intra-Poplar)	SD triplet error rate (intra-Poplar)	Average triplet error rate (Poplar-to-reference)	SD triplet error rate (Poplar-to-reference)	Average RF error rate (intra-Poplar)	SD RF error rate (intra-Poplar)	Average RF error rate (Poplar-to-reference)	SD RF error rate (Poplar-to-reference)
*Mortierellomycotina*	0.4253	0.2600	0.4677	0.0946	0.0410	0.0473	0.0470	0.0423
*Rhodophyta*	0.0340	0.0542	0.2163	0.1894	0.0760	0.0896	0.0870	0.0870

a Distances shown are from the reference and from each other upon five iterations.

**Table 6.** Triplet and RF error rates for *Puccinia*.

Input type	No. gene trees	Average triplet error rate (intra-Poplar)	SD triplet error rate (intra-Poplar)	Average triplet error rate (Poplar-to-reference)	SD triplet error rate (Poplar-to-reference)	Average RF error rate (intra-Poplar)	SD RF error rate (intra-Popular)
*Puccinia*	50	0.56	0.3761	0.62	0.1095	0.30	0.2513
*Puccinia*	100	0.28	0.3518	0.74	0.1342	0.00	0.00
*Puccinia*	500	0.28	0.3518	0.58	0.0447	0.00	0.00

**Table 7.** Time per task (two cores per worker) in building the *Kickxellomycotina* tree with 50 gene trees from unannotated genomes, averaged over five runs.

Task	Number of invocations	Average time	Total time (hh:mm:ss)
Find ORFs	13	00:00:04	0:00:55
Build BLAST database	1		0:00:04
Search BLAST	13	0:02:28	0:32:10
Make groups	1		0:00:11
Align sequences	50	0:00:09	0:07:47
Construct gene trees	50	0:00:03	0:02:51
ASTRAL-Pro	1		0:00:00.2
Other	6	0:00:00	0:00:02
Total			0:44:00

**Table 8.** Time per task (two cores per worker) in building the *Kickxellomycotina* tree with 100 gene trees from unannotated genomes, averaged over five runs.

Task	Number of invocations	Average time	Total time (hh:mm:ss)
Find ORFs	13	00:00:05	0:01:06
Build BLAST database	1		0:00:06
Search BLAST	13	0:02:28	0:31:59
Make groups	1		0:00:11
Align sequences	100	0:00:12	0:20:36
Construct gene trees	100	0:00:46	1:16:31
ASTRAL-Pro	1		0:00:00.2
Other	6	0:00:01	0:00:03
Total			2:10:32

**Table 9.** Time per task (two cores per worker) in building the *Kickxellomycotina* tree with 500 gene trees from unannotated genomes, averaged over five runs.

Task	Number of invocations	Average time	Total time (hh:mm:ss)
Find ORFs	13	0:00:06	0:01:16
Build BLAST database	1		0:00:03
Search BLAST	13	0:02:28	0:31:59
Make groups	1		0:00:13
Align sequences	500	0:00:27	3:41:24
Construct gene trees	500	0:11:00	91:42:36
ASTRAL-Pro	1		0:00:01
Other	6	0:00:00	0:00:02
Total			95:57:34

**Table 10.** Time per task (two cores per worker) in building the *Kickxellomycotina* tree with 50 gene trees from annotated genomes, averaged over five runs.

Task	Number of invocations	Average time	Total time (hh:mm:ss)
Find ORFs	13	00:00:05	0:01:02
Build BLAST database	1		0:00:04
Search BLAST	13	0:02:28	0:32:04
Make groups	1		0:00:13
Align sequences	50	0:00:10	0:08:44
Construct gene trees	50	0:00:18	0:15:04
ASTRAL-Pro	1		0:00:00.2
Other	6	0:00:00	0:00:02
Total			0:57:12

**Table 11.** Time per task (two cores per worker) in building the *Kickxellomycotina* tree with 50 gene trees from coding sequences, averaged over five runs.

Task	Number of invocations	Average time	Total time (hh:mm:ss)
Find ORFs	0		
Build BLAST database	1		0:00:05
Search BLAST	13	0:02:16	0:29:31
Make groups	1		0:00:17
Align sequences	50	0:00:06	0:05:23
Construct gene trees	50	0:00:15	0:12:10
ASTRAL-Pro	1		0:00:00.2
Other	6	0:00:00	0:00:01
Total			0:47:28

**Table 12.** Time per task (two cores per worker) in building the *Kickxellomycotina* tree with 100 gene trees from annotated genomes, averaged over five runs.

Task	Number of invocations	Average time	Total Time (hh:mm:ss)
Find ORFs	13	00:00:05	0:01:04
Build BLAST database	1		0:00:05
Search BLAST	13	0:02:27	0:31:49
Make groups	1		0:00:11
Align sequences	100	0:00:11	0:19:08
Construct gene trees	100	0:00:20	0:32:37
ASTRAL-Pro	1		0:00:00.2
Other	6	0:00:00	0:00:01
Total			1:24:55

**Table 13.** Time per task (two cores per worker) in building the *Kickxellomycotina* tree with 100 gene trees from coding sequences, averaged over five runs.

Task	Number of invocations	Average time	Total time (hh:mm:ss)
Find ORFs	0		
Build BLAST database	1		0:00:06
Search BLAST	13	0:02:16	0:29:29
Make groups	1		0:00:17
Align sequences	100	0:00:06	0:10:43
Construct gene trees	100	0:00:12	0:19:11
ASTRAL-Pro	1		0:00:00.3
Other	6	0:00:00	0:00:01
Total			0:59:46

**Table 14.** Time per task (two cores per worker) in building the *Kickxellomycotina* tree with 500 gene trees from annotated genomes, averaged over five runs.

Task	Number of invocations	Average time	Total time (hh:mm:ss)
Find ORFs	13	00:00:05	0:01:05
Build BLAST database	1		0:00:05
Search BLAST	13	0:02:28	0:31:58
Make groups	1		0:00:22
Align sequences	500	0:00:25	3:26:02
Construct gene trees	500	0:12:50	106:5:45
ASTRAL-Pro	1		0:00:02
Other	6	0:00:00	0:00:01
Total			110:57:19

### 5.4 Mortierellomycotina

*Mortierellomycotina* is another fungal classification, with 88 genomes in JGI’s MycoCosm. As with *Kickxellomycotina*, some species have multiple genomes in the database, and the tree was generated from genome assemblies, omitting annotations. In a run with 200 gene trees, as with the *Kickxellomycotina* trees, we observe that the intra-Poplar stochasticity and the Poplar-to-gold standard triplet error rates and RF error rates are comparable (Table 5). The Newick versions of the trees are included in the supplementary materials.

### 5.5 Rhodophyta

**Figure 4.**
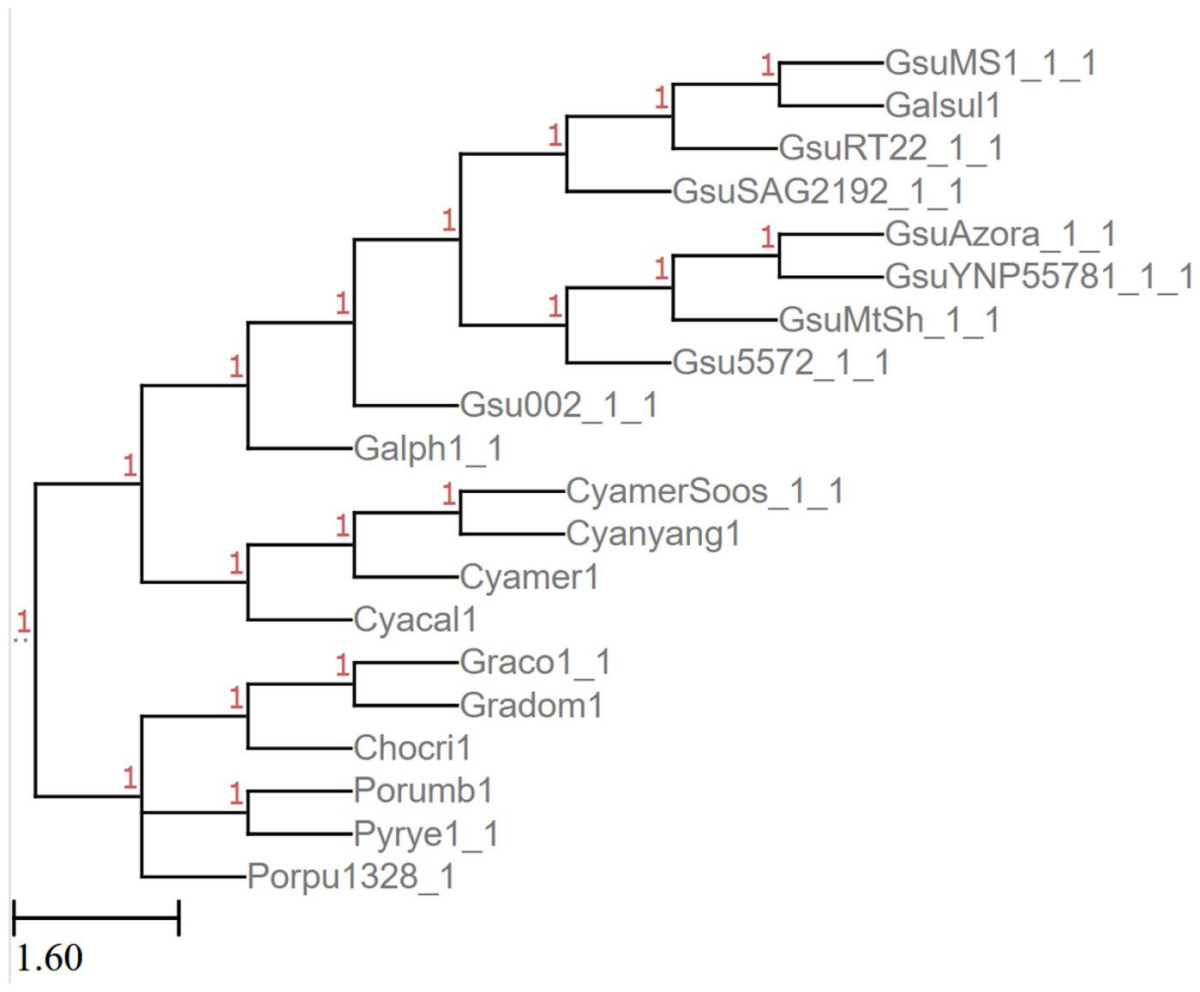
Reference *Rhodophyta* tree from JGI’s MycoCosm.

**Figure 5.**
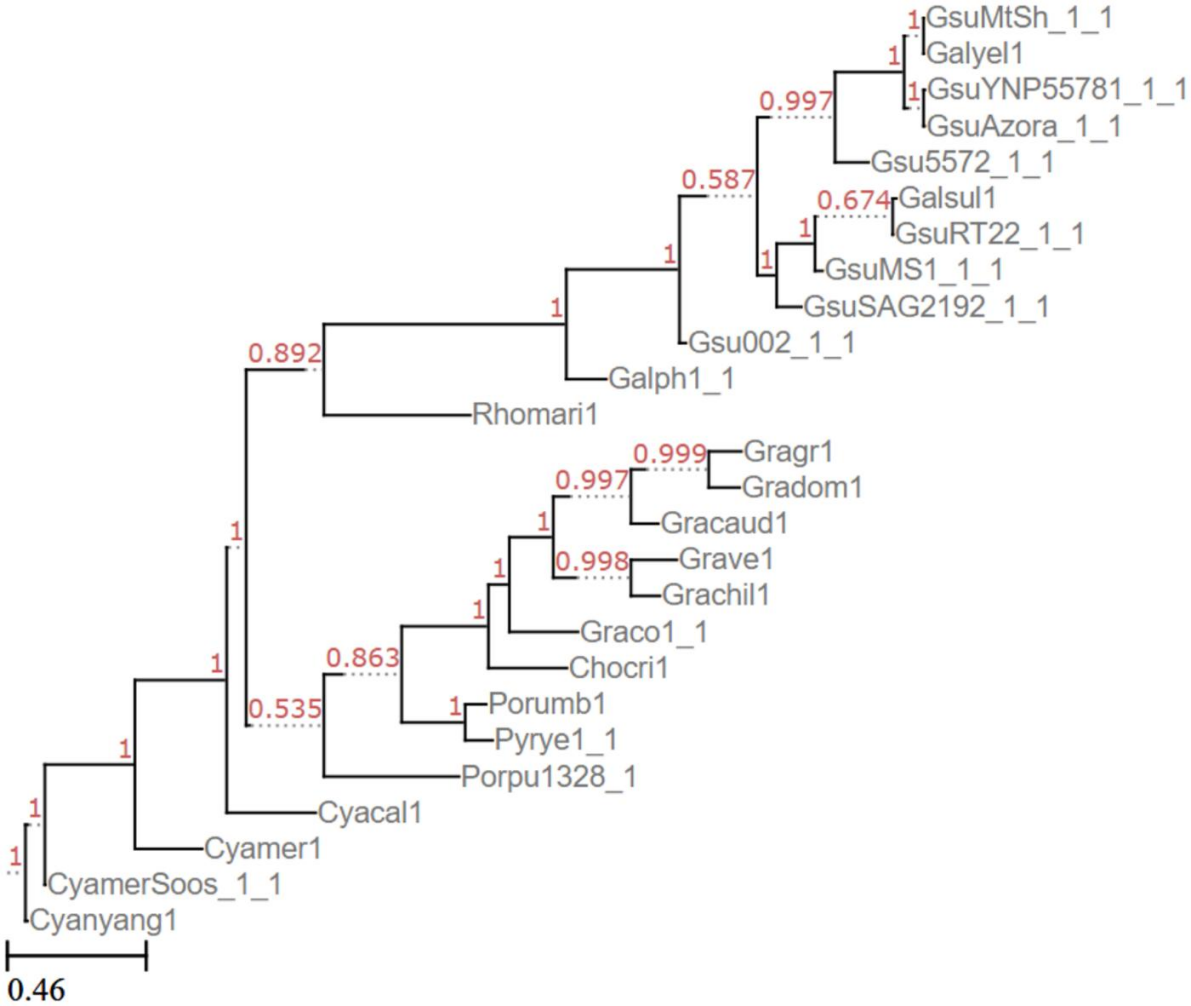
*Rhodophyta* tree generated by Poplar with 200 gene trees given coding sequences as input.

We also tested *Rhodophyta*, an algal classification with 26 genomes available through JGI’s PhycoCosm, 20 of which are included in their tree. Again, in a single run with 200 gene trees, Poplar’s RF distance from the reference tree was comparable with the intra-Poplar distance of trees across runs (Table 5). We note, however, that the triplet distances between Poplar runs is smaller than the Poplar-to-gold standard distance. This is likely due to a few differences between sets of triplets without any significant differences in overall splits, showing the importance of examining multiple distance metrics when evaluating trees. The two trees are shown in Figs. 4 and 5.

### 5.6 NCBI commonly studied organisms

**Figure 6.**
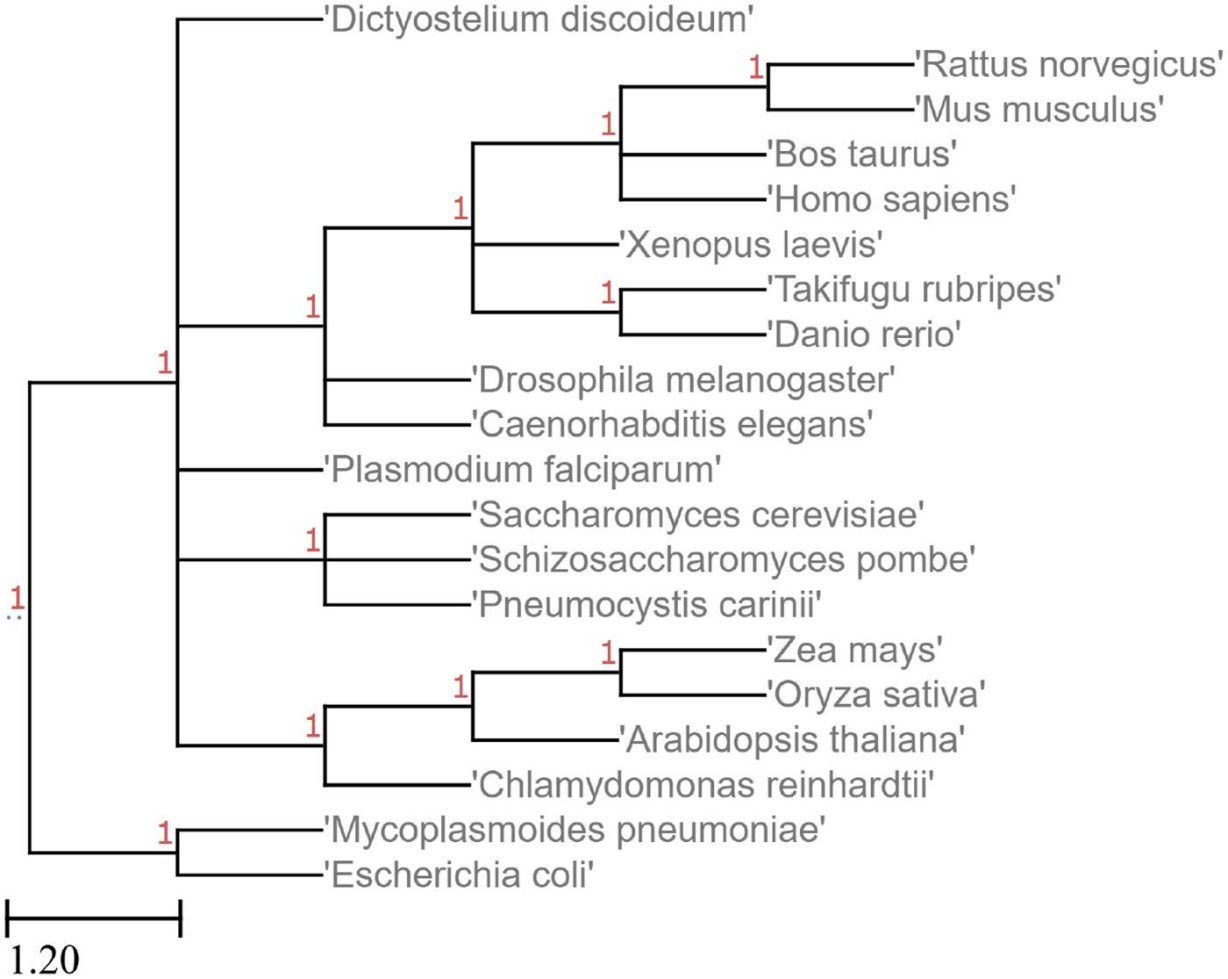
Reference tree for NCBI commonly studied organisms, from NCBI.

**Figure 7.**
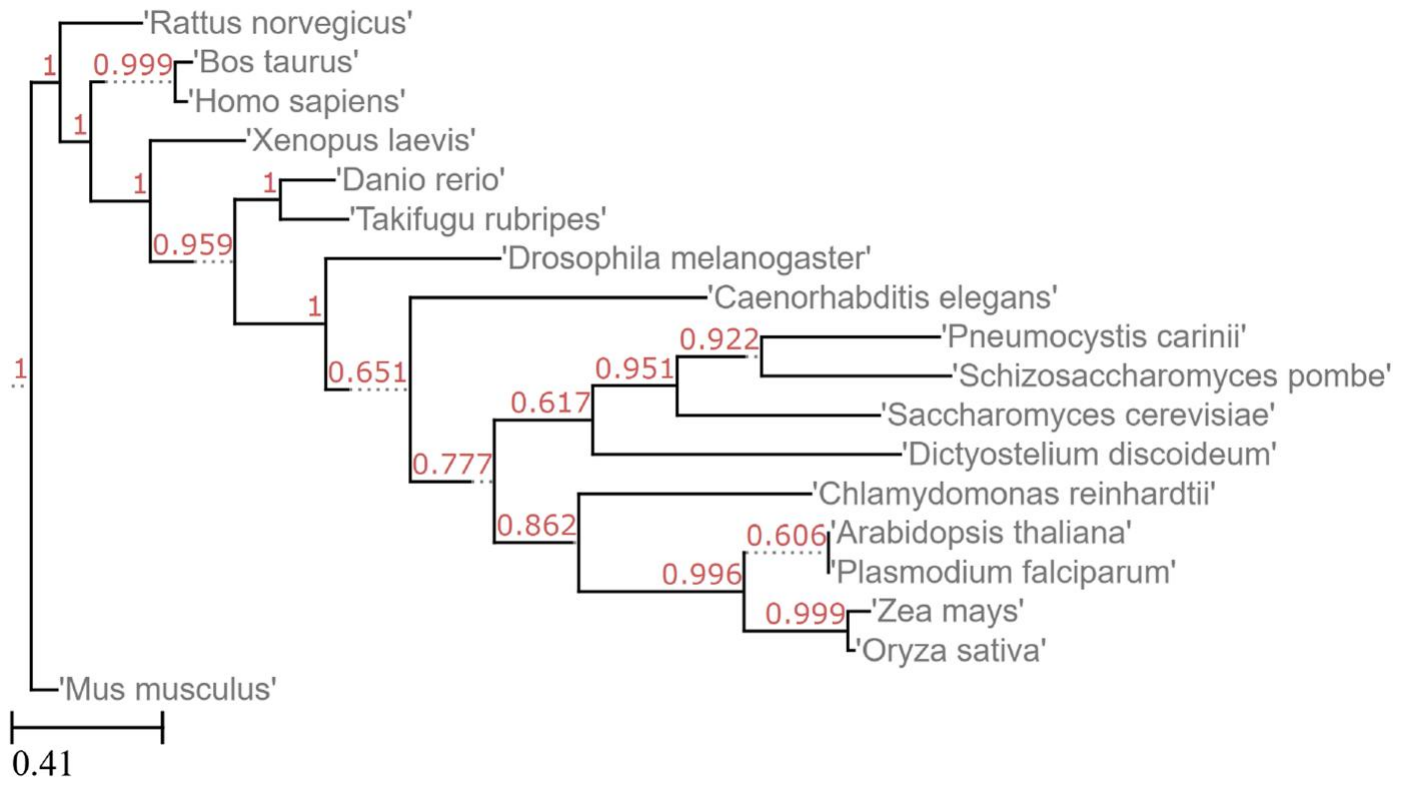
Tree generated by Poplar from the NCBI genomes given 200 gene trees and coding sequence input.

These 20 organisms from NCBI’s database were included to demonstrate the usage of Poplar on genomes outside of fungi and algae, as well as on a tree with a vast spread of species (Figs. 6 and 7).

Two runs of Poplar, both with 200 gene trees, resulted in slightly different trees. Because of the random selection of the groups, Poplar is not deterministic. Given enough gene trees, the accuracy impact of the randomness will typically be small, while the runtime impact may be significant.

The output of Poplar did not include *Mycoplasmoides pneumoniae* or *Escherichia coli* (Fig. 7). This exclusion is likely due to their distance from the other species. The other difference was in the placement of *Plasmodium falciparum*. In the first run, it was placed among the plants, leading to a Robinson-Foulds distance of 20%. In the second run, it was placed accurately next to *Dictyostelium discoideum*, and therefore the Robinson-Foulds distance was 0%. The misplacement of *P. falciparum* was most likely due to the large spread of the included species. If a species does not share as much genetic material with the other species in the tree, it will be included in fewer groups and gene trees. As a result, a misplacement in one tree that might otherwise be noise can be perpetuated into the species tree.

### 5.7 Puccinia graminis

**Figure 8.**
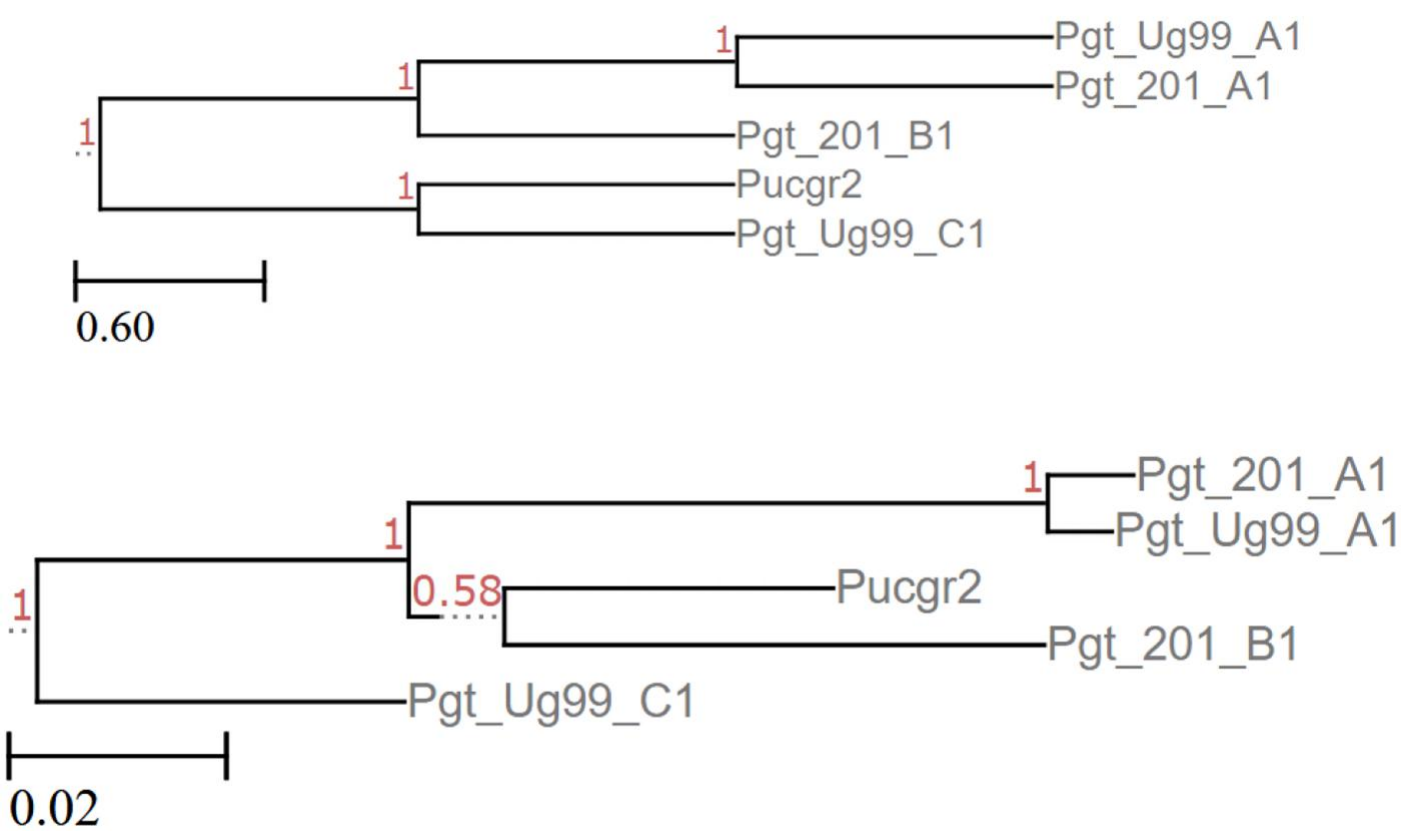
*Puccinia* graminis reference tree from MycoCosm (top) and Poplar with 100 or 500 gene trees (bottom).

The five genomes of the species *Puccinia graminis* are included to contrast the diversity of the NCBI Commonly Studied Organisms, and demonstrate the effectiveness of Poplar on genomes that are closely related. Error rates are shown in Table 6. Runs with 50 gene trees varied between each other, although the distance from the MycoCosm tree was consistent for all trees. Using 100 or 500 gene trees, all five trees were the same as each other, although they varied from MycoCosm’s tree. As shown in the Fig. 8, the two trees vary in the relationship between Pucgr2 and Pgt_201_B1. The node in MycoCosm’s tree connecting (Pgt_Ug99_A1, Pgt_201_A1) to Pgt_201_B1 has much lower support than the other nodes in the tree. While all other nodes have the maximum support of 1.0, this split has a support of only 0.2850. It is plausible that the difference between the MycoCosm and Poplar trees is supported by the available data.

### 5.8 Performance

We assessed performance on the 14 *Kickxellomycotina* genomes, using the full genomes. The tests were run on CPU nodes on a high-performance data analytics research cluster with Dual Socket Intel E5-2683v3 2.00 GHz CPUs.

In these runs, each worker uses two cores, so each node can run 28 tasks at a time. Two nodes were used per block, and Parsl was permitted a maximum of four blocks.

Tables 7 and 8 shows the performance of Poplar when used with 50 and 100 trees, respectively. The total time is the sum of the times for all processes, while the runtime is the time for the user from the submitted job being granted resources by the cluster to the time when the final tree is produced. The node time (not measured) would be between these times, approximately the total time divided by 28, as each node is divided into 28 workers.

In the same way, Table 9 shows the performance of Poplar when used with 500 trees for *Kickxellomycotina*. Again, generating the gene trees was where most of the time was spent. We also demonstrate that using annotated genomes or coding sequences results in similar performance from Poplar (Tables 7–15). We do notice that with increasing gene trees, providing annotation saves some time, which is to be expected.

**Table 15.** Time per task (two cores per worker) in building the *Kickxellomycotina* tree with 500 gene trees from coding sequences, averaged over five runs.

Task	Number of invocations	Average time	Total time (hh:mm:ss)
Find ORFs	0		
Build BLAST database	1		0:00:07
Search BLAST	13	0:02:16	0:29:29
Make groups	1		0:00:18
Align sequences	500	0:00:16	2:10:18
Construct gene trees	500	0:05:29	45:44:29
ASTRAL-Pro	1		0:00:01
Other	6	0:00:00	0:00:01
Total			48:24:42

To demonstrate the performance impact of larger genomes, we included a set of 13 *Spermatophyta* species. For each, we used the coding sequences. For two runs of Poplar, both with 500 gene trees, we averaged the performance, shown in the Table 16.

**Table 16.** Time per task (two cores per worker) in building the 13-plant tree with 500 gene trees from coding sequences, averaged over two runs.

Task	Number of invocations	Average time	Total time (hh:mm:ss)
Find ORFs	0		
Build BLAST database	1		0:00:17
Search BLAST	11728	0:00:15	47:31:40
Make groups	1		0:03:03
Align sequences	500	0:00:31	4:16:55
Construct gene trees	500	0:23:52	198:55:37
ASTRAL-Pro	1		0:00:10
Other	6	0:00:02	0:00:10
Total			250:47:50

The 13 large *Spermatophyta* genomes contrast with the 13 smaller *Kickxellomycotina* genomes, and so we can compare the *Kickxellomycotina* performance in Table 15 and the *Spermatophyta* performance in Table 16. As shown in Table 1, the average size of a *Spermatophyta* genome is far larger than that of a *Kickxellomycotina* genome, with 1G bp compared with 37M bp, and 41 415 genes compared with 8551. Because of the difference in genome sizes, the BLAST search step was split into more app invocations to increase the level of parallelism. Although the total time across two-core processes for the BLAST search was half-an-hour for the *Kickxellomycotina* case, it was 47.5 hours for *Spermatophyta*. In both cases, constructing gene trees was the most time-consuming step, and for *Spermatophyta* it required 199 hours instead of the 48.5 hours for *Kickxellomycotina*.

Because of the high level of parallelism through the capacity to run 28 processes per node and use multiple nodes, the wall time for both *Kickxellomycotina* and *Spermatophyta* was reasonable. The average wall time for the *Kickxellomycotina* workflow was 22 minutes, and the average time for the *Spermatophyta* workflow was 2 hours, with some variation due to the number of nodes available for the workflow at different points in execution. If we normalize by genome size, the ratio of compute time to size for the *Spermatophyta* is 0.2, and for *Kickxellomycotina* is 1.31, whereas, if we look at the same ratios for wall time, we see 0.12 for *Spermatophyta* versus 0.6 for *Kickxellomycotina*. What this tells us is that the software successfully scales sublinearly with increasing genome size.

**Figure 9.**
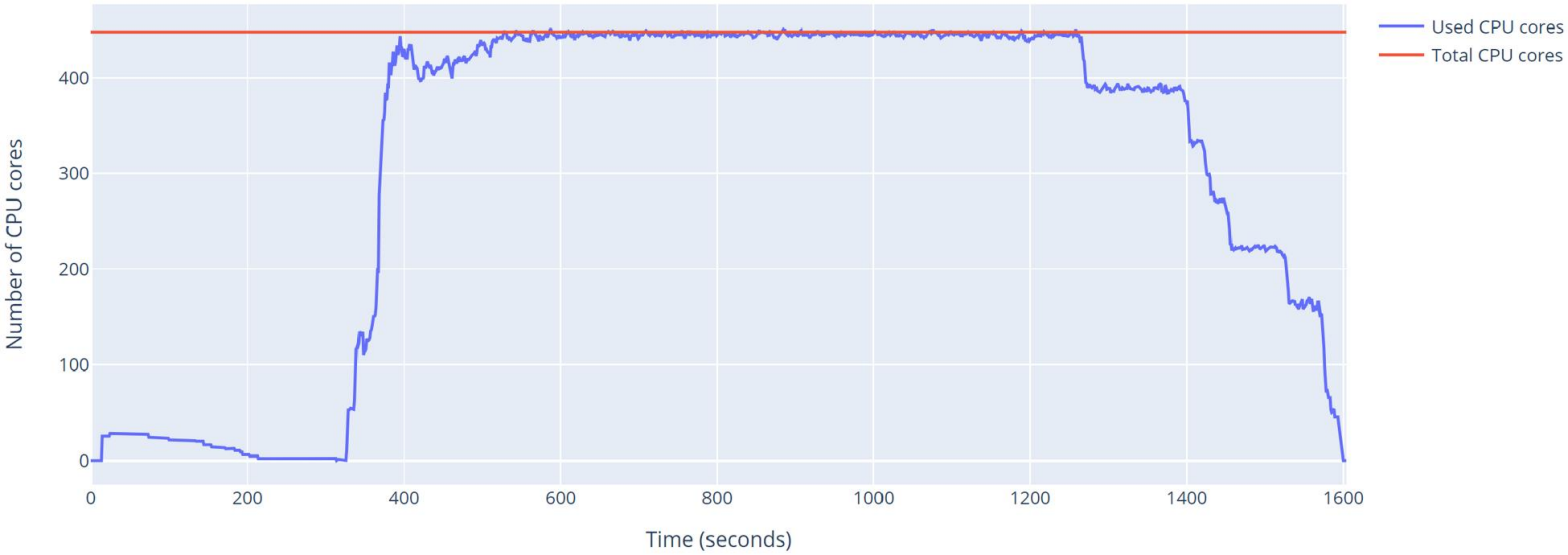
Graph of CPU usage during execution of Poplar on 14 *Kickxellomycotina* genomes using 500 gene trees.

Figure 9 shows the CPU usage throughout the run with 500 trees. During significant portion of the run, the parallelism is able to use all available CPUs.

### 5.9 Scalability

Most of the steps of Poplar are embarassingly parallel. Identification of gene sequences is a separate Parsl app for each input genome. Building the BLAST database is done with a single app, and is not currently parallelized. Searching the BLAST database for each sequence to obtain the distance matrix is parallelized through setting a threshold for the maximum number of searches per app, which is by default 50 searches. Grouping the sequences is a single process without parallelism. Executing MAFFT and RAxML for each gene group are completely available to Parsl for parallelism as their own Parsl app. Finally, ASTRAL-Pro across all gene trees is a single process, and this coordination has been a minor piece of the runtime.

Future work will build on the implementation of grouping using DBSCAN to improve efficiency and enable parallelism.

## 6 Discussion

Poplar offers a readily usable alternative to building custom phylogenomic tree pipelines, which often require subject-matter expertise, and the ability to string together multiple pieces of software that do not necessarily have compatible inputs and outputs. In addition, Poplar can utilize genomes with or without annotation, and advanced users can vary the number of gene trees that get constructed, thereby increasing the resolution of the tree. In fact, we suggest that whether increasing the gene tree number drastically changes the tree (i.e. increases the pairwise Robinson-Foulds distance, which it should not), the gene tree number should be increased until an inflection point is reached, suggesting that there is enough gene tree information for stability and trustworthiness. If necessary, methods such as the Elbow Method can be used to identify an optimal number. This higher-resolution tree will require more resources, giving users the option to choose between high resolution and speed/resource utilization.

The implementation of Poplar allows flexibility across hardware. Although testing was only performed on clusters using Slurm, Parsl supports cloud computing and other cluster management systems. Shifting between systems across workflows or within a single workflow can be done in a configuration file, with Parsl offering examples and support for a wide variety of architectures. Although Poplar is limited by the operating systems and hardware where the other software it depends on is able to run, some steps are portable to non-Unix machines and could be ported more widely with minimal user intervention.

Poplar’s results match closely with reference trees from accepted sources. Each step in Poplar is not new. Instead, the algorithms and software it incorporates are widely used and based on substantial theoretical and practical study. The novel aspect of Poplar is in the ways it allows a user to more easily run the entire pipeline with minimal interference while still obtaining results from state-of-the-art algorithms. Gathering the phylogenomic inference pipeline into one tool allows for coherency of use and communication.

A recent software tool is Reference-free Orthology-free Annotation-free discordance aware Estimation of Species (ROADIES) tree (Gupta *et al.* 2025), which uses a workflow manager to automate the phylogenomic pipeline. ROADIES introduces a novel methodology of selecting genes, and it offers the user multiple options to balance accuracy and performance. Although random sampling of sequences independent of annotation is an interesting novel method for phylogenomic tree construction, the limitations of this method across the tree of life are not fully understood, and Poplar uses annotated gene-based methods that have precedent in the literature, whether it be the utilization of annotated genes or using orfipy which identifies ORFs (Vallender 2009, Patané *et al.* 2018, Singh and Wurtele 2021). In addition, the authors present limited information about the parallelization of ROADIES, and because Poplar uses Parsl instead of Snakemake, the potential for parallelization is more flexible and dynamic, and more suited for high-performance computing. We would, however, encourage readers to critically evaluate whether their needs are more suited to the use of Poplar or ROADIES, particularly whether using prior knowledge to assign genes or ORFs is applicable, and whether they want more dynamic parallelization.

### 6.1 Limitations and future work

Poplar is designed in a modular manner, so a user may replace a step with an alternative tool. Using an alternative tool will also require providing scripts to convert input or output files if they differ from the default. Future work will include options for different methods for each step, allowing the change to be done by simply changing an argument. Adding additional options for each step will allow users who want to use particular tools or models to simply select the suitable tools via the command line or a configuration file.

Most runs of Poplar are dominated by searching the custom BLAST database for similar sequences. The time spent in this step is especially pronounced when the included genomes do not have provided coding regions or annotations. When sequence selection is not narrowed to only the coding regions or annotated genes, all ORFs in the genome are included, ballooning both the search space and the number of queries. Future work will look into both narrowing the number of queries used as well as more efficiently searching the database, given the goal of obtaining clusters.

Another area of future development of Poplar is improving results with very distantly related species. When the sequence groups are selected, the current algorithm selects a random set of groups. When some species are distantly related to the main group, they may not have sequences occur in any of the groups. In that case, they will be omitted from the species tree. In the future, the selection of sequence groups will include an optimization for including all species.

## Supplementary Material

vbaf104_Supplementary_Data

## Data Availability

Poplar is available at https://github.com/sandialabs/poplar under the GPLv3 license.
